# RNA Sequencing of Hepatobiliary Cancer Cell Lines: Data and Applications to Mutational and Transcriptomic Profiling

**DOI:** 10.3390/cancers12092510

**Published:** 2020-09-03

**Authors:** Dominique Scherer, Marcela Dávila López, Benjamin Goeppert, Sanna Abrahamsson, Rosa González Silos, Igor Nova, Katherine Marcelain, Juan C. Roa, David Ibberson, Sinan U. Umu, Trine Ballestad Rounge, Stephanie Roessler, Justo Lorenzo Bermejo

**Affiliations:** 1 Institute of Medical Biometry and Informatics, University of Heidelberg, 69120 Heidelberg, Germany; scherer@imbi.uni-heidelberg.de (D.S.); gonzalez.uni.heidelberg@gmail.com (R.G.S.); Igor.Nova@uni-heidelberg.de (I.N.); 2 Bioinformatics Core Facility, University of Gothenburg, 40530 Gothenburg, Sweden; marcela.davila@gu.se (M.D.L.); sanna.abrahamsson@gu.se (S.A.); 3 Institute of Pathology, Heidelberg University Hospital, 69120 Heidelberg, Germany; Benjamin.Goeppert@med.uni-heidelberg.de (B.G.); Stephanie.Roessler@med.uni-heidelberg.de (S.R.); 4 Department of Basic and Clinical Oncology, Faculty of Medicine, Universidad ode Chile, 8380000 Santiago, Chile; kmarcelain@uchile.cl; 5 Department of Pathology, Faculty of Medicine, Millennium Institute of Immunology and Immunotherapy, Pontificia Universidad Católica de Chile, 8330024 Santiago, Chile; jcroa@med.puc.cl; 6 Deep Sequencing Core Facility, CellNetworks Excellence Cluster, University of Heidelberg, 69120 Heidelberg, Germany; david.ibberson@bioquant.uni-heidelberg.de; 7 Department of Research, Cancer Registry of Norway, 0379 Oslo, Norway; Sinan.Ugur.Umu@kreftregisteret.no (S.U.U.); Trine.Rounge@kreftregisteret.no (T.B.R.); 8 Department of Informatics, University of Oslo, 0373 Oslo, Norway

**Keywords:** hepatobiliary cancer, gallbladder cancer, exome mutations, transcriptomics

## Abstract

**Simple Summary:**

Research on gallbladder cancer (GBC) has been largely neglected and molecular GBC data is underrepresented in public databases. Cancer cell lines constitute a valuable tool to examine the mechanisms of malignant transformation and identify potential therapeutic targets. Here we use RNA sequencing to characterize 23 commercial hepatobiliary cancer cell lines, including ten GBC cell lines, and provide detailed mutation and gene expression data to the research community. We illustrate the practical utility of the released information by (1) assessing the presence of specific mutations in the investigated cancer cell lines, (2) comparing global gene expression patterns in cell lines and primary biliary tumours and (3) examining the expression levels of specific genes. The released data and showcase applications will ease the design of in vitro cell culture assays for future studies.

**Abstract:**

Cancer cell lines allow the identification of clinically relevant alterations and the prediction of drug response. However, sequencing data for hepatobiliary cancer cell lines in general, and particularly gallbladder cancer (GBC), are sparse. Here, we apply RNA sequencing to characterize 10 GBC, eight hepatocellular carcinoma, and five cholangiocarcinoma (CCA) cell lines. RNA extraction, quality control, library preparation, sequencing, and pre-processing of sequencing data were implemented using state-of-the-art techniques. Public data from the MSK-IMPACT database and a large cohort of Japanese biliary tract cancer patients were used to illustrate the usage of the released data. The total number of exonic mutations varied from 7207 for the cell line NOZ to 9760 for HuCCT1. Researchers planning experiments that require TP53 mutations could use the cell lines NOZ, OCUG-1, SNU308, or YoMi. Mz-Cha-1 showed mutations in ATM, SNU308 presented SMAD4 mutations, and the only investigated cell line that showed ARID1A mutations was GB-d1. SNU478 was the cell line with the global gene expression pattern most similar to GBC, intrahepatic CCA, and extrahepatic CCA. EGFR, KMT2D, and KMT2C generally presented a higher expression in the investigated cell lines than in Japanese primary GBC tumors. We provide the scientific community with detailed mutation and gene expression data, together with three showcase applications, with the aim of facilitating the design of future in vitro cell culture assays for research on hepatobiliary cancer.

## 1. Introduction

Cancer cell lines constitute a valuable tool to examine the mechanisms involved in malignant transformation and to inspect and predict drug response [1]. Immense efforts were recently undertaken to achieve molecular characterization of hundreds of cancer cell lines derived from a large variety of tissues [2,3]. Mutation, DNA methylation, copy number alteration, and gene expression data are publicly available in databases such as the Catalog of Somatic Mutations in Cancer (COSMIC) and the Cancer Cell Line Encyclopedia (CCLE), with the latter combining molecular profiles of 1457 cell lines with drug response data, allowing the identification of clinically relevant oncogenic alterations [2,3].

Cancer types that are common in low- and middle-income countries but relatively rare in industrialized regions, such as gallbladder cancer (GBC), are underrepresented in the existing databases. For example, the largest in vitro drug-screening database, which contains drug response data for more than 700 compounds tested on 1691 well-characterized cell lines derived from 41 different tissues, includes data for only three GBC cell lines [4]. The limited availability of public omics data hampers efficient design of in vitro models and the development of novel therapeutic approaches.

Hepatobiliary cancer comprises hepatocellular carcinoma (HCC; International Classification of Diseases, 10th Revision Code (ICD-10) C22.0), intrahepatic cholangiocarcinoma (iCCA; ICD-10 C22.1), extrahepatic cholangiocarcinoma (eCCA; ICD-10 C24.0), and GBC (ICD-10 C23). Worldwide, hepatobiliary cancer represents the second leading cause of cancer-related death (ICD-10 C22: 781,631 deaths, ICD-10 C23–24: 165,087 deaths), with strong regional variation in incidence and mortality around the globe [5,6]. The aggressiveness of hepatobiliary tumors and the lack of clinically useful biomarkers for risk prediction, early detection, and targeted therapies lead to alarmingly low five-year survival rates of 5% for GBC and 10% for CCA [7,8]. While some advances were made in the field of CCA research showing promising results for several non-invasive biomarkers such as circulating tumor cells, extracellular vesicles, microRNAs (miRNAs), and metabolites, GBC remains an orphan disease [9,10].

In the work described here, we applied next-generation RNA sequencing to comprehensively characterize 23 commercially available hepatobiliary cancer cell lines (GBC *n* = 10, HCC *n* = 8, CCA *n* = 5), aiming at providing detailed mutation and gene expression data to the research community. Furthermore, we also illustrate the usefulness of the released information in three typical experiment-planning applications: (1) assessment of the presence of specific mutations in the investigated hepatobiliary cancer cell lines; (2) comparison of the global gene expression patterns in the sequenced cell lines and in primary biliary tumors; (3) examination of the expression levels of particular genes in the cell lines. We anticipate that the released data and showcase applications will facilitate the design of in vitro cell culture assays for future studies and provide a valuable resource to the community.

## 2. Results

### 2.1. General Sequencing Statistics

We found the coverage patterns typical for RNA sequencing experiments and gene body coverage consistent with minimum RNA degradation (Figure S1a,b). The number of raw reads in the investigated cell lines varied from 29.3 million (SNU478) to 75.6 million (HuCCT1; Table S1). The percentage of mapped reads ranged from 93% (HepG2) to 96% (YoMi). After filtering by read depth, allelic depth, mapping quality and Fisher strand, the total number of exonic mutations (single-nucleotide variants (SNVs) and insertion/deletions (indels)) varied from 7200 (NOZ) to 9751 (HuCCT1) (Table S3a,b). Most of the annotated mutations were located in the 3′ untranslated region (UTR) and exonic regions (Figure S1c). Approximately 40% of the identified exonic variants might impact gene function (Figure S1d).

### 2.2. Presence of Specific Mutations in the Investigated Cell Lines

Our first showcase application considered the choice of an appropriate cell line for an in vitro experiment based on the presence or absence of particular mutations. To illustrate the utility of the released data, we exemplarily analyzed the genes most frequently mutated in primary GBC tumors according to the MSK-IMPACT database and the study by Li et al. [11,12,13,14]. Among the 18 primary GBC tumors in MSK-IMPACT, at least 10% showed one or more mutations in *TP53, ATM, SMAD4, ARID1A, ARID1B, CTNNB1, KEAP1, NF1, NOTCH3,* and *PTPRD*. The exact mutation percentages are shown in the head of Table 1. G-415 presented no mutation in any of the selected genes (Table 1). Researchers planning in vitro experiments that require *TP53* mutations for a GBC study could use NOZ, OCUG-1, SNU308, or YoMi. *ATM* showed mutations in Mz-Cha-1, *SMAD4* presented mutations in SNU308, and the only investigated cell line that featured *ARID1A* mutations was GB-d1. SNU308 showed one or more mutations in *TP53, SMAD4,* and *KEAP1.*

With the exception of *TP53* and *NF1*, the mutation percentages in GBC cell lines and in primary MSK-IMPACT GBC tumors differed appreciably. *TP53* mutations were observed in 40% of the GBC cell lines and 44% of the primary GBC tumors, while *NF1* mutations were found in 10% of the GBC cell lines and 11% of the primary GBC tumors. *ARID1B, CTNNB, NOTCH3,* and *PTPRD* were mutated in primary MSK-IMPACT GBC tumors, but showed no mutation in the investigated GBC cell lines. *KEAP1* and *NF1* showed mutations in some of the investigated HCC cell lines, while *ARID1B* and *NF1* showed one or more mutations in the investigated CCA cell lines.

In the 32 GBC tumors analyzed by Li et al., *TP53, ZNF521, KMT2C, ERBB2,* and *ERBB3* showed the highest mutation rates (Table S2) [11]. With the only exception of one synonymous mutation in *KMT2C* in the GBC cell line Mz-Cha-1, the above-mentioned mutations in *TP53, ZNF521, KMT2C, ERBB2,* and *ERBB3* were not mutated in any of the investigated GBC cell lines (Table S2).

**Figure 1:**
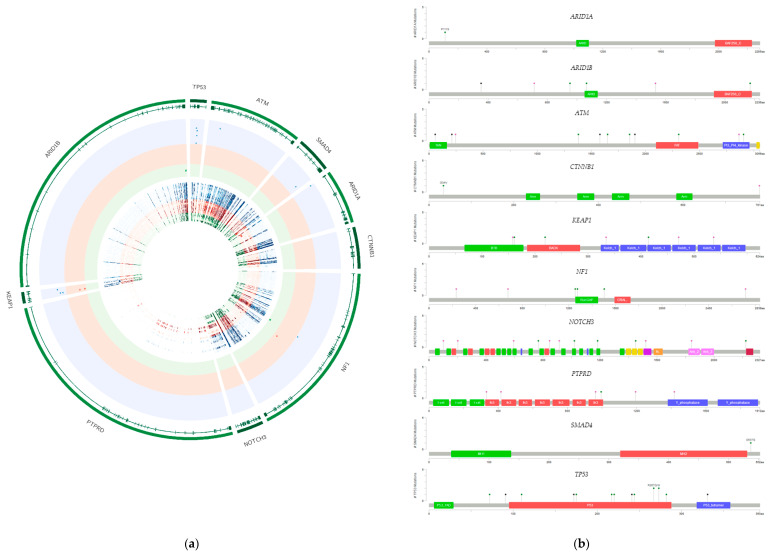
Circos plot of exonic mutations in the investigated hepatobiliary cancer cell lines (panel A) and their visualization using MutationMapper (panel B). (**a**) The tracks in the Circos plot show (1) the names of the genes with a mutation frequency of at least 10% in primary GBC tumors from MSK-IMPACT, (2) the genomic structure (exons for all potential isoforms from Ensembl are included), (3) the detected exonic mutations with at least 20× coverage, with GBC, HCC, and CCA cell lines depicted in blue (mutations = triangles), red (mutations = circles), and green (mutations = squares), respectively, and (4) sequencing coverage, where darker colors indicate increasing sequencing depth, with GBC in blue, HCC in red, and CCA in green. (**b**) Identified mutations in genes with a mutation frequency of at least 10% in primary GBC tumors according to MSK-IMPACT are depicted using MutationMapper (green: missense mutations, black: truncating mutations, red: in-frame mutations, violet: fusion mutations, pink: any other mutation type).

Additional information on the complete list of identified mutations including amino-acid changes, together with SIFT (Sorting Intolerant from Tolerant) and PolyPhen-2 pathogenicity predictions, is displayed in Table S3c. For example, after exclusion of single-nucleotide polymorphisms, all four identified *TP53* mutations were found in GBC cell lines (blue triangles and blue circle in Figure 1a). They included two stop-gain mutations, one frameshift deletion, and one non-frameshift deletion (Figure 1b and Table S3c). *KEAP1* presented mutations in GBC and HCC cell lines (blue circle and orange circle, respectively, in Figure 1a and MutationMapper in Figure 1b), and TGBC1 and TGBC2 shared the nonsynonymous variant that translated into the amino acid change p.E219Q (damaging according to SIFT and probably damaging according to PolyPhen-2; Table S3c).

### 2.3. Global Gene Expression Patterns of the Investigated Cell Lines

**Figure 2:**
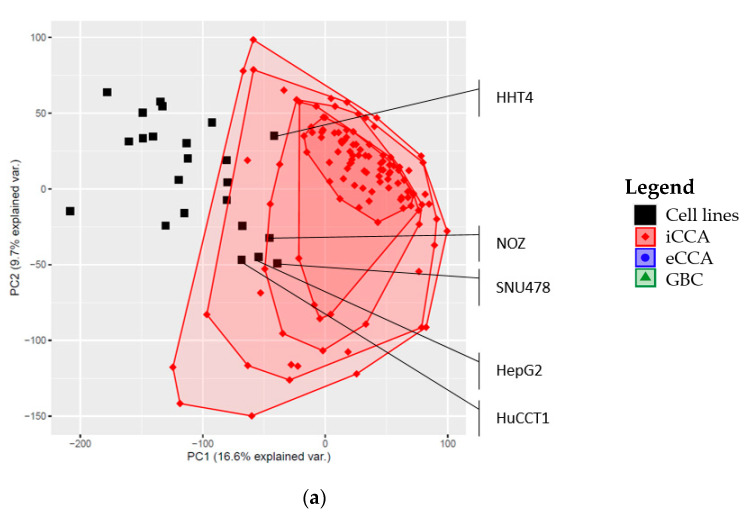

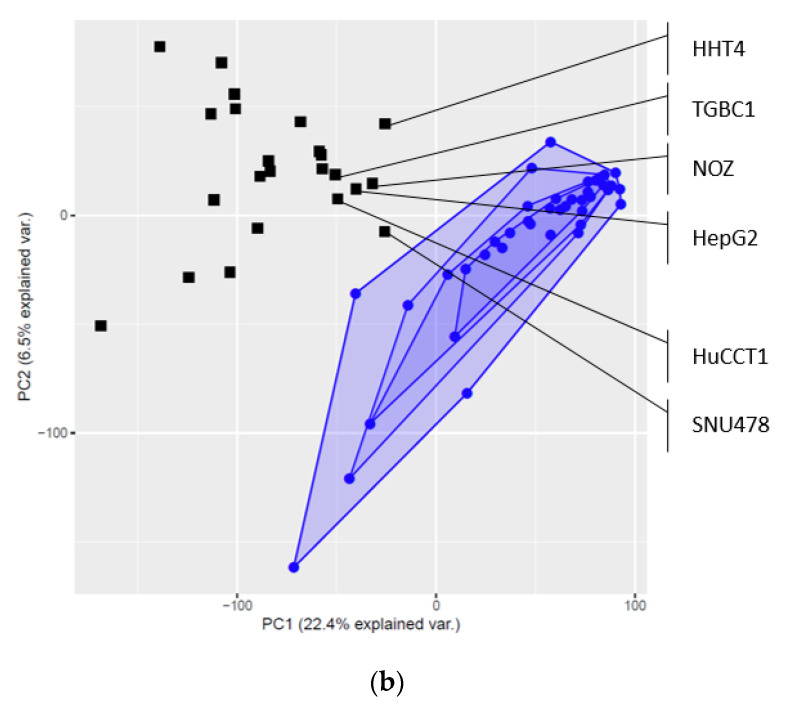

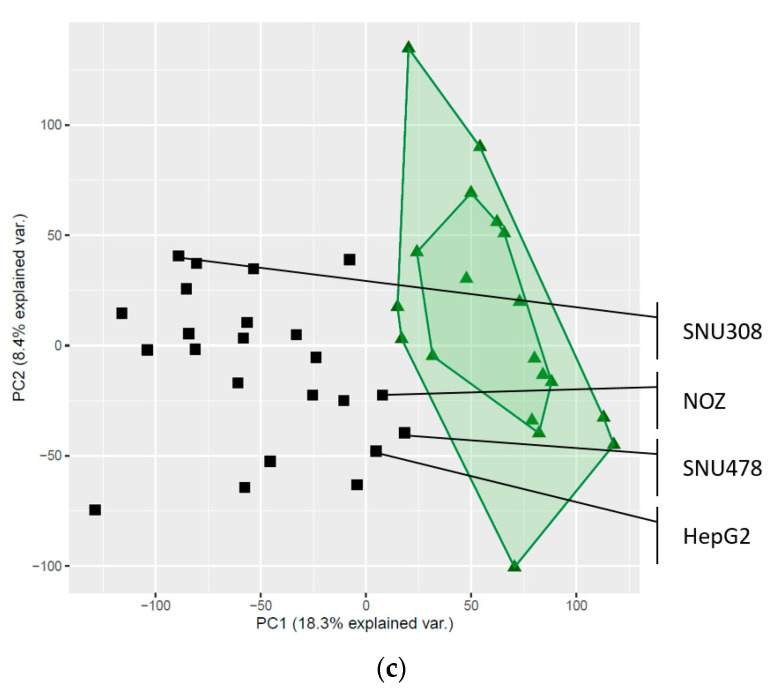
Principal component analysis (PCA) plots based on the combined gene expression data for the investigated cell lines and Japanese iCCA, eCCA, and GBC tumors. (**a**) PCA and bagplots with 100%, 90%, 70%, and 50% of the iCCA samples; (**b**) PCA and bagplots with 100%, 90%, 70%, and 50% of the eCCA samples; (**c**) PCA and bagplots with 100% and 60% of the GBC samples.

Researchers could also be interested in cell lines with global gene expression profiles similar to those of a given collection of primary tumors. To illustrate the use of the released data, we considered the sets of primary Japanese GBC (*n* = 19), eCCA (*n* = 40), and iCCA (*n* = 121) with RNA sequencing data investigated by Nakamura et al. [15]. Figure 2 shows the results from a joint principal component analysis (PCA) of gene expression data from the investigated cell lines and the Japanese tumors. Figure 2a depicts the bagplots (bivariate version of the traditional, univariate boxplots) for iCCA. The outermost area contains 100% of the tumor samples, while the second outermost area contains 90% of the tumor samples; more internal areas include 70%, 60%, and 50% of the samples (corresponding to the univariate quantiles). According to this plot, the cell lines with the global gene expression patterns most similar to iCCA were SNU478, NOZ, and HepG2. Figure 2b shows the PCA and corresponding bagplots for eCCA. Here, SNU478 was the cell line with a global gene expression pattern most similar to Japanese eCCA. Figure 2c shows the corresponding results for GBC tumors. The lower number of samples for this type of tumor resulted in just two areas with 100% and 60% of the samples. The cell lines NOZ, SNU478, and HHT4 showed the global gene expression pattern most similar to primary Japanese GBC tumors (closest to the center of the innermost area), followed by HepG2 (Figure 2c).

We also considered small RNA sequencing data, which were available for nine out of the 10 characterized GBC cell lines, to assess the robustness of the results shown in panel C of Figure 2. The median correlation between gene expression measurements using RNA and small RNA sequencing for the nine GBC cell lines was rho = 0.76. The correlation was lowest for SNU308 (rho = 0.69) and highest for GB-d1 (rho = 0.79, please see Figure S2), suggesting a good consistency between RNA and small RNA expression measurements. The PCA plots based on RNA and small RNA sequencing data confirmed that, among the nine investigated GBC cell lines, NOZ consistently showed the global gene expression pattern most similar to primary Japanese GBC tumors (closest to the center of the innermost area in Figure S3a,b). This application demonstrates the usefulness of gene expression profile analyses to select cell lines most similar to specific set of samples.

### 2.4. Expression Levels of Specific Genes in the Investigated Cell Lines

In our third and last showcase application, we examine the expression of particular genes in the investigated gallbladder cancer cell lines. We exemplarily consider 12 genes previously reported to be altered in at least 10% of primary GBC tumors from Japanese patients (*TP53, TERT, ARID2, EGFR, CCND1, CCND3, ERBB2, KMT2D, KMT2C, TET1, TET2, TET3*) [15]. We quantified gene expression levels for both cell lines and GBC tumors as transcripts per million (TPMs); the median, and the fifth and 95th TPM percentiles, in Japanese GBC samples are shown in the head of Table 2 and Table S4. TPM gene expression values for the investigated cell lines are depicted in red (higher expression than primary GBC), green (lower expression than primary GBC), and black (expression similar to primary GBC). Gene expression values shown in the upper part of the table rely on RNA sequencing. The lower part of the table shows values generated using small RNA sequencing. The consistency between the categories assigned to the gene expression of the cell lines (higher than, similar to, or lower than in primary Japanese GBC tumors) based on RNA vs. small RNA sequencing is shown in the last row (median concordance rate 61%, 100% concordance for *EGFR, KMT2D,* and *KMT2C*).

Researchers planning in vitro experiments that require *TP53* overexpression could use OCUG-1 and should not use TGBC1 or TGBC2. Researchers planning in vitro experiments that require *TERT* expression levels consistent with the average expression observed in Japanese primary GBC tumors could use Mz-Cha-1. Most genes and particularly *EGFR, KMT2D,* and *KMT2C* presented a higher expression in the investigated cell lines than in the GBC tumors; *ERBB3* was somewhat of an exception. TPM expression values from RNA sequencing for all genes and all investigated cell lines are provided in Table S5.

## 3. Discussion

Cancer cell lines are valuable tools for researchers to investigate and identify mechanisms of cancer development and treatment success. In recent years, the (epi)genetic features of hundreds of cell lines were characterized, and the data were made publicly available, generating resources that greatly facilitate tumor modeling and drug screening. However, the existing resources focus mainly on common cancer types and do not support research into cancers such as GBC, considered an orphan disease.

The present study provides mutation and transcriptome data for hepatobiliary cancer cell lines, with a special focus on GBC. We illustrate the scientific potential of the generated data with three showcase applications: (1) assessment of the presence of specific mutations in the investigated hepatobiliary cancer cell lines, (2) comparison of global gene expression patterns in the sequenced cell lines and in primary biliary tract cancers, and (3) examination of the expression levels of particular genes in the cell lines.

As part of our first application, we considered genes that are commonly mutated in primary GBC tumors and compared their mutation frequencies with those in the investigated hepatobiliary cancer cell lines. We noticed that, with the exception of *NF1* and *TP53*, the mutation frequencies differed appreciably between GBC cell lines and primary GBC tumors. This also applies to three recently characterized GBC cell lines of Chilean origin, in which none of the genes most frequently altered in MSK-IMPACT and the study by Li et al. (heads of Table 1 and Table S2) showed mutations [18]. We also compared the mutations identified in the present study with the existing data for the selected genes in the CCLE, the COSMIC Cell Lines Project and the study by Klijn et al. [2,16,17]. The CCLE provides mutation information for only two GBC, seven HCC, and three CCA cell lines, while COSMIC and the study by Klijn et al. included even fewer cell lines. Perfect matches between our data and the CCLE datasets were observed for mutations in *KEAP1* and *SMAD4* (Table 1). We also observed some discrepancies between our generated data and the datasets from CCLE, COSMIC, and the study by Klijn et al. In particular, *TP53* mutations were more frequently observed in the CCLE compared to our data. The discrepancies between public databases in mutation detection is a well-known phenomenon which may point to inadequate sequencing of GC-rich areas of the exome, variation in SNP filtering, or acquisition/loss of mutations [19]. Specifically, the inconsistency of *TP53* mutations found in presumably the same cell line is a known issue in cell line research, often caused by cross-contamination or misidentification [20]. To rule out both sources of errors, the cell lines used in our study were carefully tested for identity and contamination. Another reason for the different mutations observed in our data compared to CCLE, COSMIC, and the study by Klijn et al. could be the use of different sequencing techniques. As we applied RNA sequencing, our data are restricted to mutations located on transcripts that are expressed in the investigated cell lines. Cross-references between different data sources are crucial for a reliable examination of mutation spectra in cancer cell lines.

The global gene expression profile of GBC tumors was well reflected by NOZ, which is indeed classified as a GBC cell line [21]. NOZ and SNU478 also presented global gene expression profiles that were similar to iCCA and eCCA, respectively. Other cell lines that showed similar gene expression profiles with iCCA and eCCA tumors were HuCCT1, an iCCA cell line, HepG2, which was originally misclassified as HCC, but is derived from hepatoblastoma, and HHT4, which is an hTERT-immortalized cell line derived from primary hepatocytes [21,22,23]. TGBC1, a cell line that is derived from gallbladder cancer, showed a global gene expression pattern more similar to iCCA and eCCA than to GBC [21]. The global gene expression of the three Chilean cell lines showed different patterns compared to the commercial cell lines (Figure S3c).

These analyses show that the origin of the tissue from which cell lines are derived does not necessarily reflect the tumor feature of interest. Accessible information on the mutation and transcriptome characteristics of the investigated cell lines is crucial for the design of in vitro experiments. The resources provided as part of this study will facilitate future research on hepatobiliary cancer, and particularly on GBC.

Based on the expression of distinct genes, cell lines can be selected for the analyses of specific pathways. Most of the selected genes were overexpressed in GBC cell lines compared with tumors. However, for most genes, there was at least one GBC cell line with similar expression levels to GBC tumors. The exceptions were *EGFR, KMT2D,* and *KMT2C*, which were always expressed more highly in GBC cell lines than in GBC tumors. The gene expression of the selected genes in the cell lines of Chilean origin was higher compared to the primary tumor samples with few exceptions (Table S6) [18].

## 4. Materials and Methods

### 4.1. Characterized Hepatobiliary Cancer Cell Lines, Contamination Testing, and RNA Extraction and Sequencing

We investigated 10 GBC cell lines (G-415 (also known as TKG0642), GB-d1, Mz-Cha-1, NOZ, OCUG-1, OZ, SNU308, TGBC1 (also known as TGBC1TKB), TGBC2 (also known as TGBC2TKB), YoMi), eight HCC cell lines (Hep3B, HepG2, HHT4, HLE, HLF, HuH1, HuH6, HuH7), and five CCA cell lines (EGI-1, HuCCT1, KMCH, SNU478, TFK-1) (Table S7) [21,22,24]. All cell lines were regularly tested to be negative for mycoplasma contamination using MycoAlert (Lonza, Basel, Switzerland) and authenticated by short tandem repeat analysis.

G-415, SNU308, HepG2, HuCCT1, SNU478, and TFK-1 cells were cultured in RPMI-1640 medium; GB-d1, Mz-Cha-1, OCUG-1, TGBC1, TGBC2, YoMi, HLE, HLF, HuH1, HuH6, HuH7, EGI-1, and KMCH were cultured in DMEM medium; Hep3B was cultured in MEM medium; OZ and NOZ cells were cultured in William’s E medium. All media were supplemented with 10% fetal bovine serum (Thermo Fisher Scientific, Offenbach, Germany) and 1% penicillin–streptomycin (100 IU/mL and 100 g/mL). EGI-1 was additionally supplemented with l-glutamine and non-essential amino acids. All media and supplements were obtained from Sigma-Aldrich (Taufkirchen, Germany). The immortalized hepatocyte cell line HHT4 was provided by Curtis C. Harris and cultured as previously described [24,25]. Cell lines were cultivated at 37 °C in a humidified 5% CO_2_ atmosphere.

Total RNA was extracted from cells with NucleoSpin RNA Kit (Macherey-Nagel, Düren, Germany) according to the manufacturer’s protocol. RNA integrity was determined using the Agilent RNA Nano 6000 chips on the Agilent Bioanalyzer 2100 system (Agilent Technologies, Palo Alto, CA, USA). In total, 1.5 µg of RNA was used to prepare RNA sequencing libraries. The libraries were generated using the NEBNext Ultra II Directional RNA Preparation Kit (New England Biolabs, Ipswich, MA, USA) in conjunction with the NEBNext Poly A Selection Module, and the NEBNext Multiplex Oligo’s for Illumina. Libraries were quantified on the Qubit (Thermo Fisher, Offenbach, Germany) with the High Sensitivity DNA Assay and quality-checked with the DNA 1000 Chip on the Agilent Bioanalyzer. Using these values, samples were equimolar pooled and sequenced on the Illumina HiSeq 2500 (Illumina, San Diego, CA, USA) in 125-bp paired-end mode.

### 4.2. Quality Control, Pre-Processing, and Statistical Analysis of RNA Sequencing Data

FastQC version 0.11.2 [26] was used to assess the quality of raw reads, which were subsequently filtered using PRINSEQ [27] version 0.20.3 with the following quality parameters: -min_qual_mean 20, -ns_max_p 10, -trim_qual_right 3, -min_len 30. Variant calling was performed following the GATK guideline for RNA sequencing data, which includes two-pass alignment with STAR (2.5.2b) [28], removal of PCR duplicates with Picard tools (2.1.0) [29], and variant calling with GATK [30] 3.5. The GRCh38.90 version of the human genome was used as reference. The mapping quality and coverage distribution were assessed with Qualimap (2.2.1) [31]. The threshold values for mutation detection were set at read depth >20, allelic depth >10, mapping quality >40, and Fisher’s strand <60. Variants were annotated with Annovar, and only exonic variants were used for downstream analyses [32]. A Circos plot was used to depict the gene location of the identified mutations and the sequencing coverage [33]. Gene expression values were quantified as TPMs using the output from featureCounts of the Subread package version 1.6.4 [34]. The R language and environment for statistical computing was used for principal components analysis (PCA) of gene expression data and the representation of the two first principal components with superimposed “bagplots”, which were obtained using the package “depth” [35].

RNA sequencing data of all investigated cell lines are deposited [36].

### 4.3. External Data Used for the Exemplary Applications

To illustrate the utility of the released mutation data, we considered the list of genes most frequently mutated in primary GBC tumors according to the MSK-IMPACT database and the study by Li et al. [11,12]. We included only primary gallbladder tumors from the MSK-IMPACT Clinical Sequencing Cohort (MSKCC, Nat Med 2017) (*n* = 19) and excluded the second sample from one patient, who contributed with two samples, resulting in *n* = 18 MSK-IMPACT samples in total. The study by Li et al. comprised *n* = 32 samples. We also compared the global gene expression profiles of the investigated cell lines with the expression patterns of the collection of primary Japanese GBC, eCCA, and iCCA provided by Nakamura et al. [15,37]. Finally, we examined the expression of genes previously reported to be altered in at least 10% of primary GBC tumors from Japanese patients (Figure 5 in the article by Nakamura et al. [15]) in the investigated cell lines.

### 4.4. Validation of Gene Expression Results

We also analyzed small RNA sequencing data, which were available for nine out of the 10 characterized GBC cell lines, to examine the robustness of results described in our second and third showcase applications. Small RNA sequencing was performed using NEBNext^®^ Small RNA Library Prep Set for Illumina (Cat. No E7300, New England Biolabs, Ipswich, MA, USA) with a cut size on the pippin prep (Cat. No CSD3010, Sage Science, Beverly, MA, USA) covering RNA molecules from 17 to 47 nucleotides, which enables capture of messenger RNA (mRNA) fragments as shown previously [38]. Libraries were sequenced on the HiSeq2500 (Illumina, San Diego, CA, USA) to reach an average depth of 18 million total reads per sample. Total reads were trimmed for adapters using AdapterRemoval v2.1.7 and mapped to the human genome (hg38) using Bowtie2 v2.2.9. HTSeq was used to count the reads mapped to mRNA exons in GENCODE v26 using an established bioinformatics workflow [38], and read counts were transformed to transcripts per million (TPM). The robustness of gene expression findings was assessed by (1) the correlation between gene expression values generated using RNA and small RNA sequencing, (2) the similarity/disparity of PCA plots based on RNA vs. small RNA sequencing data for the GBC cell lines, and (3) the consistency between the categories assigned to the nine GBC cell lines (higher than, similar to, or lower than expression in primary GBC tumors) for 12 genes previously reported to be frequently altered in tumors from Japanese patients (*TP53, TERT, ARID2, EGFR, CCND1, CCND3, ERBB2, KMT2D, KMT2C, TET1, TET2, TET3*) based on RNA vs. small RNA sequencing data for the GBC cell lines.

## 5. Conclusions

Based on the data generated in this study, researchers will be able to select cell lines that best reflect their experimental requirements from a selection of GBC, CCA, and HCC cell lines, all of which are available from commercial distributors. The RNA sequencing data described here will facilitate further studies into GBC, an orphan disease not only in research but also in public databases.

## Figures and Tables

**Table 1:** Mutations in hepatobiliary cancer cell lines detected by RNA sequencing. GBC—gallbladder cancer; HCC—hepatocellular carcinoma; CCA—cholangiocarcinoma.

Cell Line		Gene																																							
		Proportion of Primary GBC Tumours in MSK-IMPACT with One or More Mutations																																							
Type	Name	*TP53*				*ATM*				*SMAD4*				*ARID1A*				*ARID1B*				*CTNNB1*				*KEAP1*				*NF1*				*NOTCH3*				*PTPRD*			
		44%				33%				28%				22%				11%				11%				11%				11%				11%				11%			
		A	B	C	D	A	B	C	D	A	B	C	D	A	B	C	D	A	B	C	D	A	B	C	D	A	B	C	D	A	B	C	D	A	B	C	D	A	B	C	D
GBC	G-415																																								
	GB-d1													x																											
	Mz-Cha-1					x																																			
	NOZ	x																																							
	OCUG-1	x																																							
	OZ																																								
	SNU308	x	x							x	x															x	x														
	TGBC1		x	x																						x	x	x												x	
	TGBC2																									x															
	YoMi	x																												x											
HCC	Hep3B							x																																x	
	HepG2						x														x																				
	HHT4																																								
	HLE		x	x												x																x									
	HLF		x																																						
	HuH1		x	x	x																					x	x	x	x												
	HuH6		x	x																			x	x						x										x	
	HuH7		x	x	x											x				x						x	x					x									
CCA	EGI-1		x	x				x																																	
	HuCCT1		x	x															x																						
	KMCH																	x																							
	SNU478		x																x																						
	TFK-1																													x											

Cells with colored background indicate data availability for the respective cell line in the specific database/project. Columns A: data from the present study (green); B: data from the Cancer Cell Line Encyclopedia (CCLE) (yellow); C: data from the Catalog of Somatic Mutations in Cancer (COSMIC) Cell Lines Project (blue); D: data provided by Klijn et al., 2014 (red) [2,16,17].

**Table 2:** Expression of particular genes in the investigated gallbladder cancer cell lines using RNA sequencing and small RNA sequencing. TPM—transcripts per million.

		Gene-Specific Median TPM Expression Value											
Gene Name		*TP53*	*TERT*	*ARID2*	*EGFR*	*CCND1*	*CCND3*	*ERBB3*	*KMT2D*	*KMT2C*	*TET1*	*TET2*	*TET3*
Median TPM		4.95	0.04	0.67	1.45	45.04	1.73	33.08	6.16	0.68	0.04	0.42	0.42
5th percentile–95th percentile		2.34–7.55	−0.1–0.17	0.44–0.89	0.71–2.19	6.02–84.06	0.79–2.66	30.23–34.71	3.49–8.82	0.42–0.94	−0.04–0.11	0.17–0.9	0.37–0.9
RNA Sequencing	GB-d1	14.09	1.59	1.78	16.76	184.35	2.88	39.69	34.67	1.27	0.05	1.22	1.43
	Mz-Cha-1	29.98	0.14	1.37	13.55	100.15	5.06	57.79	41.35	3.43	0.04	0.59	2.19
	NOZ	3.90	0.30	0.59	7.32	151.95	3.15	1.98	21.87	1.49	0.06	0.26	1.03
	OCUG-1	25.41	2.01	0.64	21.78	30.05	2.91	31.73	10.56	3.85	0.10	0.57	0.56
	OZ	39.80	0.36	1.84	19.97	149.99	2.85	198.17	69.93	3.18	0.06	1.12	4.59
	SNU308	41.07	0.16	-	21.38	201.48	1.67	35.15	56.81	2.62	0.11	1.93	2.51
	TGBC1	1.62	3.67	0.79	4.19	81.09	2.26	31	20.34	2.22	0.26	0.53	1.19
	TGBC2	1.50	9.54	0.51	6.35	106.40	7.28	3.16	19.03	1.53	0.11	0.80	1.58
	YoMi	4.71	0.30	3.20	24.72	256.11	6.54	284.89	66.87	5.01	-	2.20	8.11
small RNA Sequencing	GB-d1	2.07	0.45	5.25	49.02	83.05	2.53	9.65	37.48	3.35	0.58	2.13	10.45
	Mz-Cha-1	2.58	0.14	5.06	37.27	39.53	2.33	15.43	39.12	10.45	1.07	1.39	9.86
	NOZ	0.79	-	3.82	28.94	94.33	3.19	0.34	26.37	5.09	1.27	0.71	8.12
	OCUG-1	8.61	2.66	3.64	122.31	27.62	11.73	18.83	37.79	24.93	5.39	3.74	3.67
	OZ	7.29	0.31	9.89	53.12	132.32	5.01	25.38	54.95	8.71	0.71	2.25	47.57
	SNU308	2.10	0.23	0.20	17.52	51.47	1.21	2.61	23.03	2.87	-	0.81	5.73
	TGBC1	1.06	2.99	3.94	26.63	75.96	4.78	12.90	57.30	12.75	9.97	1.62	14.45
	TGBC2	0.70	6.54	2.86	16.29	60.75	6.23	0.58	34.57	3.86	0.53	0.53	10.22
	YoMi	1.03	0.43	10.73	30.21	150.31	13.48	68.40	52.66	15.80	0.86	2.93	30.39
% concordance		33	78	44	100	44	67	22	100	100	11	56	89

The color of the numbers compares the gene expression in primary Japanese GBC tumors and GBC cell lines using RNA or small RNA sequencing. Red: increased expression, green: decreased expression, black: Similar expression; the yellow background indicates concordance between RNA and small RNA sequencing comparisons.

## Supplementary Materials

The following are available online at https://www.mdpi.com/2072-6694/12/9/2510/s1: Figure S1. RNAseq coverage and mutation distribution of the investigated hepatobiliary cancer cell lines; Figure S2. Correlation between RNA and small RNA sequencing gene expression; Figure S3. PCA plots based on the combined gene expression data for the investigated cell lines and Japanese GBC tumors; Table S1. Sequencing data statistics and predicted mutations per sample; Table S2. Mutations in hepatobiliary cancer cell lines detected by RNA sequencing - Genes selected based on Li et al.; Table S3. Number of exonic mutations per gene in 23 cell lines; Table S4. Expression of particular genes in the investigated hepatobiliary cancer cell lines; Table S5. TPM (transcript per million) expression values in 23 cell lines; Table S6. Expression of particular genes in Chilean gallbladder cancer cell lines using RNA-Sequencing; Table S7. Overview of cell lines.
